# Transsplenic tract closure after transsplenic portalvenous access using gelfoam-based tract plugging

**DOI:** 10.1186/s42155-023-00383-w

**Published:** 2023-07-17

**Authors:** Meine TC, Kretschmann N, Yerdelen SS, Wacker FK, Meyer BC, Hinrichs JB

**Affiliations:** Institute for Diagnostic and Interventional Radiology, Hannover Medical School, Carl-Neuberg-Straße 1, D-30625 Hannover, Germany

**Keywords:** Gelfoam, Embolization, Splenic access, Transsplenic, Portal hypertension, Portal vein thrombosis, Cavernous transformation, Portal vein reconstruction, Transjugular intrahepatic portosystemic shunt, Variceal embolization, Portal vein stent

## Abstract

**Background:**

To assess the feasibility and safety of a gelfoam torpedo plugging technique for embolization of the transsplenic access channel in adult patients following transvenous portal vein interventions.

**Materials and methods:**

Between 09/2016 and 08/2021, an ultrasound guided transsplenic portalvenous access (TSPVA) was established in twenty-four adult patients with a 21-G needle and 4-F microsheath under ultrasound guidance. Afterwards, sheaths ranging from 4-F to 8-F were inserted as needed for the procedure. Following portal vein intervention, the splenic access tract was embolized with a gelfoam-based tract plugging (GFTP) technique. TSPVA and GFTP were performed twice in two patients. Patients’ pre-interventional and procedural characteristics were analyzed to assess the feasibility and safety of the plugging technique according Cardiovascular and Interventional Radiological Society of Europe (CIRSE) classification system. Values are given as median (minimum;maximum). Subgroup analysis of intercostal vs. subcostal puncture site for TSPVA was performed using the two-sided Mann–Whitney-U test or Student’s t-test and Fisher’s exact test. Level of significance was *p* < 0.05.

**Results:**

The study population’s age was 56 (29;71) years and 54% were female patients. Primary liver disease was predominantly liver cirrhosis with 62% of the patients. Pre-interventional model for end-stage liver disease score was 9 (6;25), international normalized ratio was 1.15 (0.86;1.51), activated partial thromboplastin time was 33s (26s;52s) and platelet count was 88.000/µL (31.000;273.000/µL). Ascites was present in 76% of the cases. Craniocaudal spleen diameter was 17cm (10cm;25cm). Indication for TSPVA was assisted transjugular intrahepatic portosystemic shunt placement in 16 cases and revision in two cases, portal vein stent placement in five cases and variceal embolization in three cases. TSPVA was successfully established in all interventions; interventional success rate was 85% (22/26). The splenic access time was 33min (10min;133min) and the total procedure time was 208min (110min;429min). Splenic access was performed with a subcostal route in 11 interventions and with an intercostal route in 15 interventions. Final sheath size was 4-F in 17 cases, 5-F in three cases, 6-F in five cases, 7-F in two cases and 8-F in one case. A median of two gelfoam cubes was used for GFTP. TSPVA- and GFTP-related complications occurred in 4 of 26 interventions (15%) with a subcapsular hematoma of the spleen in two patients (CIRSE grade 1), access-related infection in one patient (CIRSE grade 3) and both in one patient (CIRSE grade 3). In detail, one access-related complication occurred in a patient with subcostal TSPVA (CIRSE grade 1 complication) and the other three complications occurred in patients with intercostal TSPVA (one CIRSE grade 1 complication and two CIRSE grade 3 complication) (*p* = 0.614). No patient required interventional or surgical treatment due to puncture tract bleeding.

**Conclusion:**

Gelfoam-based plugging of the puncture tract was feasible and safe for transsplenic access in adult patients undergoing percutaneous portal vein interventions. The lack of major bleeding complications and complete absorption of the gelatine sponge make it a safe alternative to transjugular and transhepatic access and re-interventions via the splenic route.

## Introduction

Direct access to the portal venous system is increasingly used in a broad variety of indications [1–4]. It is established with an ultrasound guided percutaneous puncture of an intraparenchymal splenic vein branch and generation of an intraparenchymal tract to the splenic vein by insertion of an access sheath with a hemostatic valve [1–3]. This transsplenic portalvenous access (TSPVA) facilitates the reconstruction of the portal vein (PVR) with or without transjugular intrahepatic portosystemic shunt (TIPS) placement in patients with portal vein occlusion (PVT) [1–3]. Furthermore, the transsplenic route is feasible for variceal embolization (VE) of portosystemic collateral pathways in patients not eligible for TIPS placement or for portal vein stent (PVS) implantation due to portal vein stenosis or compression [5–8]. Major risk of transsplenic interventions is severe bleeding through the transsplenic portalvenous tract or even splenic rupture [9]. These substantial hazards may depend on different factors, e. g. coagulation status of the patients and puncture site related issues like sheath size or closure technique [10]. Different closure techniques of transsplenic accesses are described in the literature. In 1997, Liang et al. reported one of the first case series on splenic access for diagnostic angiography with an 18-G needle and 4-F catheter using 2 to 4 pieces of gelfoam injected via a 1ml tuberculin syringe with an overall complication rate of 29% (5/17) and bleeding complications requiring blood transfusion in 11% (2/17) probably due to incomplete sealing of the tract as the small syringe might apply uncontrollable high pressure during gelfoam application [11]. Since then, TSPVA is increasingly used with access sheaths ranging between 4-F and 9-F [5, 6, 8, 12, 13]. Various closure techniques of the transsplenic tract have been described with permanent embolic agents including lipiodol-/N-butyl-cyanoacrylate-embolization or coil embolization with or without use of lipiodol ethiodized oil or gelfoam [5, 6, 8, 12, 13]. The reported complication rates of these techniques varied between 0 and 20% and the complication profile consisted predominantly of splenic bleeding [5, 6, 8, 12, 13]. As patients potentially need repeated interventions via the transsplenic route a permanent embolization of the splenic access site might not be favorable. Therefore, gelfoam, a non-permanent embolic agent, might be an appropriate alternative for sufficient closure of TSPVA [14–17]. Thus, the purpose of this study was to analyze the feasibility and safety of a gelfoam-based tract plugging (GFTP) technique for embolization of the TSPVA after percutaneous portal vein interventions in adult patients.

## Materials and methods

### Study population

The Picture Achieving Computational System and Radiology Information System was screened for patients undergoing TSPVA between 09/2016 and 02/2021. Overall, 26 portal vein interventions in 24 adult patients with TSPVA and GFTP were identified comprising the study population. This retrospective study was approved by the institutional Human Subject’s Review Board and was in accordance with the 1964 Declaration of Helsinki and its later amendments or comparable ethical standards.

### Splenic access closure technique

Portal vein interventions via a splenic access were routinely performed after paracentesis and during general anesthesia on dedicated angiography systems (Artis Q® or Artis Pheno® Siemens Healthineers, Erlangen, Germany) by two board-certified interventional radiologists (J. B. H. and B. C. M.). TSPVA was achieved via an intercostal or a subcostal puncture of a splenic vein branch with a 21-G needle of a micropuncture set (4-F Custom Procedure Kit, Merit Medical, Merit Medical Systems, Inc. South Jordan, Utah, USA) under ultrasound guidance. The 21-G needle was removed and a 4-F microsheath/dilator was placed over a short 0.018-inch (4-F Custom Procedure Kit, Merit Medical, Merit Medical Systems, Inc. South Jordan, Utah, USA) in the transsplenic tract and intraparenchmal splenic vein branch. Then, the 4-F microsheath was exchanged by a 4-F up to 8-F access sheath with hemostatic valve according to the procedure needs (Avanti + , Cordis, Waterloo, Belgium).

**Fig. 1:**
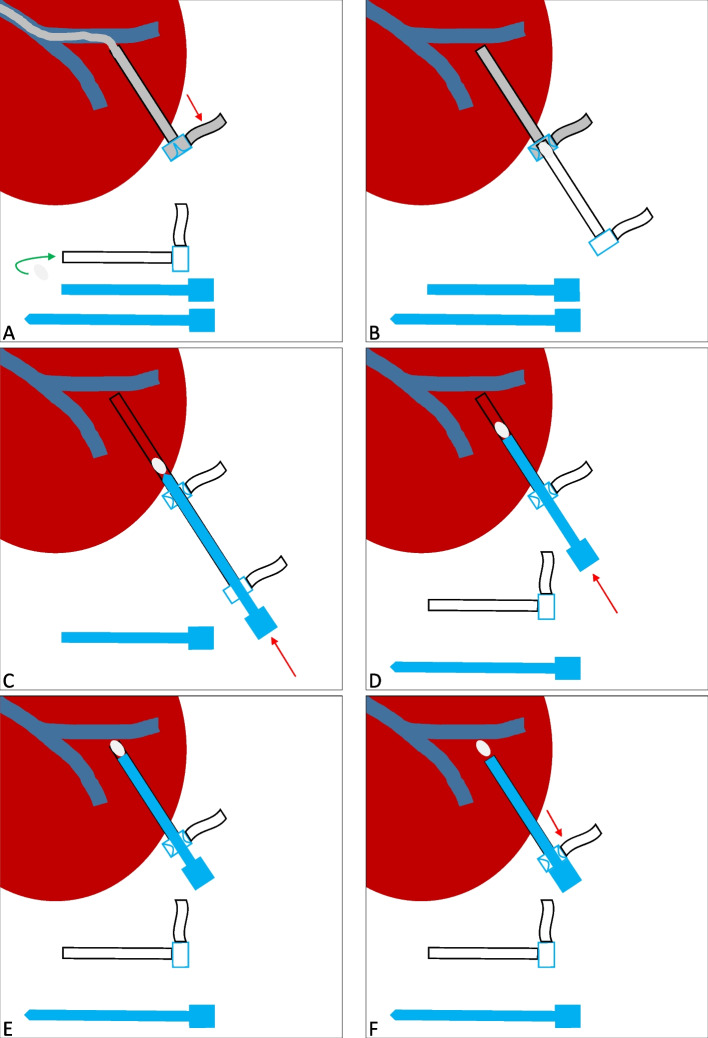
**A** Contrast media was injected through the access sheath transsplenic tract and intraparenchymal splenic vein branch in the patient while the access sheath was pulled backwards (red arrow) until a parenchymal tract is visible. Then, a gelfoam plug (gray) was loaded in the tip of a second sheath, the insertion sheath (green arrow). Of note, two dilators were shown in this image (blue). One dilator was shortened concisely to the end of the sheath with an incision scalpel, the insertion dilator. **B** Then, the tip of the gelfoam-loaded insertion sheath was introduced through the membrane of the access sheath in the splenic tract (both sheaths were the same size). **C** Pushing the normal length dilator into the insertion sheath (red arrow), we transferred the gelfoam plug in the access sheath. **D** After successful transfer of the gelfoam plug in the access sheath, the insertion sheath and the normal length dilator were removed. The insertion dilator was introduced in the access sheath to advance the gelfoam plug. **E** The insertion dilator is almost loaded in the access sheath. **F** Finally, the access sheath is gently pulled backward (red arrow) while the insertion dilator is held in position to release the gelfoam plug in the tract (“withdrawal technique”)

After the intervention, the TSPVA was closed using the GFTP technique. The technique of GFTP is depicted in a schematic illustration in Fig. 1. First, the interventional devices were removed, but the access sheath was left in the transsplenic tract and intraparenchymal splenic vein branch. Then, the access sheath located within the splenic vein branch was pulled back until splenic parenchyma enhanced under fluoroscopy using contrast media. Meanwhile, an insertion sheath and dilator for gelfoam application was prepared by the assistant physician. Therefore, the dilator of a second access sheath of the same size was shortened concisely to the end of the sheath using a conventional incision scalpel. This second sheath and shortened dilator was named insertion sheath/dilator. Thereafter, a gelfoam cube (Gelita, B. Braun, Melsungen, Germany) was cut and formed to plugs fitting into the tip of the insertion sheath. One gelfoam plug was placed in the tip of the insertion sheath outside the patient. The tip of the gelfoam-loaded insertion sheath was then introduced through the membrane of the access sheath in the splenic tract. Pushing the normal length dilator into the insertion sheath, we transferred the gelfoam plug in the access sheath in the patient. The insertion sheath and the normal length dilator were removed and the insertion dilator was introduced in the access sheath in the patient to advance the gelfoam plug to the tip of the access sheath. Due to the shortening of the insertion dilator the gelfoam plug will reach the puncture tract at the time the insertion dilator is completely loaded into the sheath. If a normal length dilator is used, there will be the risk of misplacement of the gelfoam plug in the splenic vein and portal system. This uncontrolled release of the gelfoam plug with the normal length dilator can be avoided using the shortened dilator, referred in this manuscript as insertion dilator. Of note, when the insertion dilator is almost loaded into the access sheath, we pull the access sheath backward while the insertion dilator is held in position to release the gelfoam plug in the tract (“withdrawal technique”). This procedure is repeated until the access sheath left the splenic capsule.

### Data collection and analysis

Patient characteristics included age, gender and primary disease. Pre-interventional characteristics such as model for end-stage liver disease (MELD), international normalized ratio (INR), activated partial thromboplastin time (aPTT) (s), platelet count (PLT) (per µL), administration of anticoagulation and transfusion products and spleen diameter were assessed. Procedural characteristics were indication for splenic access, success rate of splenic access and the intervention, splenic access time (min), total procedure time (min), splenic access route (subcostal versus intercostal), sheath size (F), number of gelfoam cubes needed for tract embolization and access-related complications. Splenic access time was defined as the time from the begin of the percutaneous needle propagation to the final puncture of the splenic vein confirmed by contrast media or successful positioning of a guidewire in the splenic vein branch as described before [18]. Total procedure time was the time from preparation of the sterile field until the patient was transferred out of the angiography suite to the intensive care unit, intermediate care unit or general ward. Access-related complications such as splenic hematoma, intraperitoneal hemorrhage, access-related infections and death were recorded according the Cardiovascular and Interventional Radiological Society of Europe (CIRSE) classification system [19]. Complications were assessed with fluoroscopic imaging during the intervention or in follow-up examination of the patients with physical examination, abdominal ultrasound, abdominal computed tomography and blood tests during hospital stay [18, 20]. Data were collected from interventional imaging, interventional reports, medical transfer or medical discharge reports. Values were given as median (minimum;maximum). Since the complexity of the access route might be different, a subgroup analysis was performed between interventions with intercostal and subcostal route for TSPVA. Data distribution was analyzed using the Shapiro–Wilk test. Test of homogeneity was conducted with the Levene’s test. Comparisons were performed using the Mann–Whitney-U or Student’s t-test and Fisher’s Exact test dependent on data distribution. Level of significance was *p* < 0.05. Statistical analysis was conducted using commercially available software (SPSS Statistics, Version 28, IBM, New York).

## Results

### Study population

Overall, 26 TSPVA were established in 24 patients. Median age of the patients was 56 years and 54% of the patients were female. Primary disease was cirrhotic in 15 patients and non-cirrhotic in 9 patients. In detail, the etiology of portal hypertension was ethyltoxic (*n* = 8) or cryptogenic (*n* = 1) liver cirrhosis, liver fibrosis (*n* = 1), secondary sclerosing cholangitis (*n* = 1), post-transplantation fibrosis (*n* = 1), portal vein thrombosis (*n* = 4), cavernous transformation of the portal vein (*n* = 3), post-operative portal vein stenosis after resection of cholangiocellular carcinoma (*n* = 3), pancreatic carcinoma (*n* = 1) and portal vein obstruction due to focal nodular hyperplasia (*n* = 1). Median pre-interventional MELD score was 9 (6;25). In 10 cases of 26 portal interventions (38%), patients received pre-interventional anti-aggregation (acetylsalicylic acid *n* = 1) or prophylactic anticoagulation therapy (tinzaparin *n* = 4, dalteparin *n* = 2, enoxaparin *n* = 2 and fondaparinoux *n* = 1). Blood products (pack of red blood cells, platelet or prothrombine complex concentrates) were administered in seven different patients prior the intervention (26%); two patients of these seven patients were additionally on anti-aggregation/-coagulation therapy (one patient with acetylsalicylic acid and another patient with tinzaparin medication). Median pre-interventional INR was 1.15, median pre-interventional aPTT was 33s and median pre-interventional PLT was 88.000/µL. Ascites was present in 76% (20/26) of the cases addressed by pre-interventional paracentesis if possible. The pre-interventional spleen diameter showed a median of 17cm. Detailed characteristics were tabulated in Table 1.

**Table 1:** Patient, pre-interventional and procedural characteristics

Patient characteristics	
Number of patients	24
Age (years)	56 (29;71)
Gender (male / female)	11 / 13
Liver disease (cirrhotic / non-cirrhotic)	15 / 9
Ethyltoxic liver cirrhosis	8
Cryptogenic liver cirrhosis	1
Liver fibrosis	1
SCC	1
Post-LTx	1
PVT	4
CTPV	3
CCC	3
PCA	1
FNH	1
**Pre-interventional characteristics**	
Number of interventions	26
MELD	9 (6;25)
INR	1.15 (0.86;1.51)
aPTT (s)	33 (26;52)
PLT (1*100/uL)	88 (31;273)
Anti-aggregation or -coagulation pre-interventionally (no. of patients)	10
Transfusion pre-interventionally (no. of patients)	7
Ascites pre-interventionally (present / none)	20 / 6
Spleen diameter (cm)	17 (10;25)
**Procedural characteristics**	
Number of interventions	26
Indication	
TIPS placement*	16
TIPS revision	2
PVS	5^
VE	3°
Splenic access success rate (successful / unsuccessful)	26 / 0
Interventional success rate (successful / unsuccessful)	22 / 4
Splenic access time (min)	33 (10;133)
Total procedure time (min)	208 (110;429)
Splenic access route (subcostal / intercostal)	11 / 15
Sheath size (F)	4 (4;8)
No. of gelfoam cubes	2 (1;5)
Clinical significant complications (no. of complications)	4

*Abbreviaion*: *aPTT* activated partial thromboplastin time, *INR* International normalized ratio, *FNH* Focal nodular hyperplasia, *MELD* Model for end-stage liver disease, *PLT* Platelet count, *PCA* Pancreatic carcinoma, *post-LTx* Post-transplantation liver fibrosis, *PVT* Portal vein thrombosis, *CTPV* Cavernous transformation of the portal vein, *CCC* Cholangiocellular carcinoma, *TIPS* Transjugular intrahepatic portosystemic shunt, *PVS* Portal vein stent, *VE* Variceal embolization and *SSC* Secondary sclerosing cholangitis

^*^In one case an extrahepatic portosystemic shunt was established between liver and portal vein

^In one case no portal vein stent was placed, because the postoperative stenosis of the portal vein did not increase the portal pressure > 12 mmHg as expected from pre-interventional computed tomography. °In one case no embolization was performed, because no active bleeding and no potential feeders from the portal system could be identified

Focused on patients with access-related complications, there was no difference in the age, gender distribution, liver disease, MELD, aPTT, INR, PLT, ascites and spleen diameter compared to the total study cohort. In contrast, the number of patients receiving anti-coagulation or anti-aggregation therapy prior the portal vein intervention was relatively high with three of four patients in the group with access-related complications (75%). In consideration of subgroup analysis, the patient data were not significant different, but the pre-interventional spleen diameter was larger in the subgroup with the subcostal TSPVA than the subgroup with intercostal TSPVA (p_spleen_diameter_ = 0.008). Patient and pre-interventional data were given in Table 2 and 3.

**Table 2:** Patient and procedural characteristics of interventions with complications

Intervention Number	1	2	3	4
**Patient characteristics**				
Age (years)	42	52	62	62
Gender	female	female	male	female
Liver disease (cirrhotic / non-cirrhotic)	PVT (cirrhotic)	CTPV (non-cirrhotic)	Cryptogenic (cirrhotic)	CCC (non-cirrhotic)
**Pre-interventional characteristics**				
MELD	18	10	25	5
INR	1.51	1.31	1.15	0.86
aPTT (s)	52	31	31	28
PLT (1*100/uL)	31	40	86	146
Anti-aggregation/-coagulation pre-interventionally	Dalteparin, 10000IE, 1/d	Fondaparinux, 2.5mg, 1/d	Aspirin, 100mg, 1x/d	none
Transfusion pre-interventionally	none	none	2 pack of RBC	none
Ascites pre-interventionally	present	present	present	none
Spleen diameter (cm)	25	18	15	10
**Procedural characteristics**				
Indication	TIPS	VE”	VE	PVS
Splenic access	successful	successful	successful	successful
Interventional success	successful	successful	successful	successful
Splenic access time (min)	35	14	133	56
Total procedure time (min)	322	110	205	113
Splenic access route	subcostal	intercostal	intercostal	intercostal
Sheath Size (F)	5-F	4-F	4-F	7-F
No. of gelfoam cubes	2	2	2	2
Clinical significant complications	splenic hematoma	erysipelas, NP°	splenic hematoma, SBP^	splenic hematoma
CIRSE classification	1	3	3	1

*Abbreviations*: *aPTT* activated partial thromboplastin time, *CIRSE* Cardiovascular and interventional radiological society of Europe, *CCC* Cholangiocellular carcinoma, *CTPV* Cavernous transformation of the portal vein, *INR* International normalized ratio, *MELD* Model for end-stage liver disease, *NP* Nosocomial peritonitis, *PVS* Portal vein stent, *PLT* Platelet count, *PVT* Portal vein thrombosis, *RBC* Red blood cell, *SBP* Spontaneous bacterial infection, *TIPS* Transjugular intrahepatic portosystemic shunt, *VE* Variceal embolization

^ceftriaxone and metronidazole i.v., vancomycin and gentamicin i.p., °piperacillin/tazobactam and meropenem + daptomycin.” In this case no embolization was performed, because no active bleeding and no potential feeders from the portal system could be identified

**Table 3:** Patient, pre-interventional and procedural characteristics

	**Intercostal pathway**	**Subcostal pathway**	**p**	**Statistical test**
**Patient characteristics**				
Number of patients	15	11^		
Age (years)	61 (29;71)	43 (29;71)	0.124	MWU
Gender (male / female)	7 / 8	5 / 6	1	FE
Liver disease (cirrhotic/ non-cirrhotic)	9 / 6	7 / 4	1	FE
**Pre-interventional characteristics**				
Number of interventions	15	11		
MELD	9 (5;25)	13 (6;18)	0.259	MWU
INR	1.14 (0.86;1.45)	1.15 (1.00;1.51)	0.433	MWU
aPTT (s)	33 (26;40)	32 (26;52)	0.691	MWU
PLT (1*100/uL)	105 (40;267)	80 (31;273)	0.909	MWU
Anti-aggregation or -coagulation pre-interventionally (no. of patients)	5 / 10	5 / 6	0.689	FE
Transfusion pre-interventionally (no. of patients)	4 / 11	3 / 8	1	FE
Ascites pre-interventionally (no. of patients)	3 / 11	3 / 12	1	FE
Spleen diameter (cm)	14 (10;23)	19 (12;25)	0.008	TT (homogeneity of variance)
**Procedural characteristics**				
Number of interventions	15	11		
Indication (TIPS/ PVS or VE)	9 / 6	8 / 3	0.395	FE
TIPS placement*	8	8		
TIPS revision	1	1		
PVS	3	1		
VE	3	1		
Splenic access success rate (successful / unseccussful)	15 / 0	11 / 0	*	FE
Interventional success rate (successful / unsuccessful)	2 /13	2 / 9	1	FE
Splenic access time (min)	25 (10;133)	36 (13;121)	0.232	MWU
Total procedure time (min)	195 (110;420)	255 (110;429)	0.311	MWU
Sheath size (F)	4 (4;7)	6 (4;8)	0.003	MWU
No. of gelfoam cubes	2 (1;5)	2 (1;3)	0.511	MWU
No. of access-related complications (present/absent)	3 / 12	1 / 10	0.614	FE
CIRSE classification grade 1	1	1		
CIRSE classification grade 2	0	0		
CIRSE classification grade 3	2	0		
CIRSE classification grade 4	0	0		
CIRSE classification grade 5	0	0		
CIRSE classification grade 6	0	0		

*Abbreviations*: *aPTT* Activated partial thromboplastin time, *CIRSE* Cardiovascular and interventional radiological society of Europe, *FE* Fischer’s exact test, *INR* International normalized ratio, *MELD* Model for end-stage liver disease, *MWU* Mann–Whitney-U test, *PVS* Portal vein stent, *PLT* Platelet count, *TIPS* Transjugular intrahepatic portosystemic shunt, *TT* Student’s t-test, *VE* Variceal embolization

^In the group with subcostal pathway, two patients underwent two interventions via the splenic access route

^*^No *p*-value calculated

### Gelfoam torpedo plugging technique

The most common indication for TSPVA was TIPS placement or revision. The success rate of splenic access was 100% and in two patients re-access via the same transsplenic route after 25 and 37 days could be established without complications. The success rate of the portal vein intervention was 85%. Median splenic access time was 33min and median total procedure time was 208min. Splenic access route was subcostal in 11 cases and intercostal in 15 cases. Sheath size for splenic access was 4-F in 17 cases, 5-F in three cases, 6-F in five cases, 7-F in two cases and 8-F in one case. A median of two gelfoam cubes was required for closure of the puncture tract. Access-related complications occurred in 15% (4/26). In detail, splenic hematoma with maximum diameter < 3cm occurred in three patients. This complication was self-limiting in two patients (CIRSE grade 1) while one patient developed additional spontaneous bacterial peritonitis, which was successfully treated with intravenous administration of ceftriaxone and metronidazole and intraperitoneal administration of vancomycin and gentamicin (CIRSE grade 3). In a fourth patient, nosocomial peritonitis and erysipelas at the cuteaneous puncture site were diagnosed after splenic access which was treated successfully with intravenous application of piperacillin/tazobactam followed by meropenem and daptomycin (CIRSE grade 3). The analysis of the different access routes (intercostal vs. subcostal) showed no significant difference concerning complication rates for TSPVA (*p* = 0.614). In detail, one access-related complication occurred in a patient with subcostal TSPVA (CIRSE grade 1) and three complications occurred in patients with intercostal TSPVA (one CIRSE grade 1 complication, two CIRSE grade 3 complications). No patient required interventional or surgical treatment due to puncture tract bleeding. In consideration of the patients, who developed access-related clinical significant complications, the indications for TSPVA varied with VE in two patients, PVS implantation in one patient and TIPS placement in one patient. Of note, in the subgroup analysis, the sheath size of the group with subcostal TSPVA was larger compared to the group with intercostal TSPVA (p_sheath_size_ = 0.003). Detailed information were given in Tables 2 and 3.

## Discussion

Embolization of TSPVA using GFTP technique is effective and safe in patients undergoing transplenic portal vein interventions. The success rate of the splenic access of 100% and the interventional success of 85% are comparable to the current literature (splenic access: 89% to 100%; interventional success: 79% to 100%) [10–13, 21, 22]. The reported complication rates in the literature range from 0 to 29% related to TSPVA [5, 6, 8, 11–13]. Our complication rate of 15% is in the middle of this range. In one of the first reports dealing with a diagnostic case series, Liang et al. has published a complication rate of the splenic access of 29% when using gelfoam for tract sealing [11]. The authors injected gelfoam pieces into the splenic tract with a 1ml tuberculin syringe, which is associated with high pressure peak during gelfoam application which might lead to incomplete sealing of the tract. The complications rates of permanent embolic agents for sealing of the splenic puncture tract range from 0 to 20% [5, 6, 8, 12]. The lowest complication rate of 0% has been reported for tract embolization using a combination of coils and lipiodol or coils and glue/ethiodized oil. However, the sample size in both case series is small (≤ 11 patients) [6, 12]. Larger case series using permanent embolic agents reported complication rates of 16% for coil and gelfoam in 18 patients and 20% for lipiodol-and N-butyl-cyanoacrylate in 46 patients [5, 13]. In another cohort of 46 patients major bleeding complications, equivalent to CIRSE grade 3 occurred in 8 patients [13, 19]. Taken together, the rates of bleeding complications following transsplenic interventions seem to be comparable and independent of the agent used for tract embolization. Hence, there appears to be no advantage of permanent embolic agents over non-permanent embolic agents in the prevention of bleeding complications. In contrast, permanent embolic agents within the transsplenic tract might hinder re-interventions via a transsplenic route, which is unlikely in case of gelfoam [15, 23–25]. This is underlined by our study with two patients undergoing re-interventions 25 days and 37 days following the first transsplenic intervention via a comparable access route. Moreover, permanent embolization is associated with increased costs and permanent artifacts on cross sectional imagingin comparison to gelfoam embolization as described in the literature [26–28]. In addition, liquid embolization might cause irreversible off-target embolization to the portal venous system or potentially life-threatening pulmonary embolism in patients undergoing TIPS placement which is unlikely when using GFTP technique [29]. In pediatric patients, a gelfoam slurry technique has been described for transsplenic tract embolization with complication rates of 7% to 27% and a maximum complication grade comparable to CIRSE grade 3 in study cohorts ranging between 11 and 30 patients [10, 14, 21]. Compared to GFTP technique, where the torpedos are applied in a very controlled fashion and are well visualized, the gelfoam slurry technique has a potential risk for off-target embolization as described for liquid embolization agents.

In addition to the embolization technique, the puncture technique and the patient’s condition may affect the complication rate after transsplenic access. Complications tend to occur more often when the needle size is < 21-G or the sheath size is > 4-F [5, 11, 13]. We use a 21-G needle for the puncture of the intraparenchymal splenic vein branch and subsequently upsize the initially inserted 4-F sheath as needed. In our study, there was no association of puncture pathway (intercostal vs. subcostal) and bleeding complications. We used an intercostal puncture pathway according to the lower-risk zone recently published by Misura et al. [30]. Nevertheless, in our opinion, ultrasound-guidance and needle angulation for splenic access is more challenging in patients with normal spleen size, small intraparenchymal splenic vein branches and intercostal needle path compared to patients with splenomegaly, dilated intraparenchymal splenic vein branches and subcostal needle path.

In consideration of the patient’s condition, anti-coagulation can be associated with an increased bleeding risk after splenic access. In our study, three of four patients with access-related complications have been on pre-interventional anti-coagulation or -aggregation. This is in line with the finding of Pimpalwar et al. that bleeding risk increases in pediatric patients on anti-coagulation before transsplenic interventions [10]. Ascites is another potential risk factor for bleeding. The direct alignment of the abdominal wall and the capsule of the spleen can limit splenic bleeding from the puncture tract and may prevent subcapsular or perisplenic hematoma formation. In our study cohort, pre-interventional ascites was common and—if possible—paracentesis was performed before the intervention as described in the literature [13]. Our study has several limitations. It is a retrospective single center study with a limited number of transsplenic portal vein interventions. There is no control group using permanent embolization such as coils, plugs or glue. Finally, historic comparisons of complication profiles and rates is challenging, because the classification and reporting of complications is not standardized between existing studies and change over time [19, 31].

In conclusion, the GFTP technique is feasible and safe in adult patients with portal vein interventions. It can be favorable for re-interventions via a comparable splenic route due to the complete absorption of the gelatine sponge. Pre-interventional anti-coagulation and intercostal needle path were associated with higher complication rates.

## Data Availability

The datasets generated and/or analysed during the current study are not publicly available due to data protection regulations, but may be available under certain legal requirements upon reasonable request.

## Abbreviations

aPTT: Activated partial thromboplastin time
CIRSE: Cardiovascular and Interventional Radiological Society of Europe
CTPV: Cavernous transformation of portal vein
FE: Fisher’s Exact test
GFTP: Gelfoam-based tract plugging
INR: International normalized ratio
MELD: Model for end-stage liver disease
MWU: Mann–Whitney-U test
VE: Variceal embolization
PLT: Platelet count
PVS: Portal vein stent
PVR: Portal vein reconstruction
PVT: Portal vein thrombosis
TIPS: Transjugular intrahepatic portosystemic shunt
TSPVA: Transsplenic portalvenous access
TT: Student’s t-test
